# Astrocytic transcription factor REST upregulates glutamate transporter EAAT2, protecting dopaminergic neurons from manganese-induced excitotoxicity

**DOI:** 10.1016/j.jbc.2021.101372

**Published:** 2021-10-29

**Authors:** Edward Pajarillo, Alexis Digman, Ivan Nyarko-Danquah, Deok-Soo Son, Karam F.A. Soliman, Michael Aschner, Eunsook Lee

**Affiliations:** 1 Department of Pharmaceutical Sciences, Florida A&M University, Tallahassee, Florida, USA; 2 Department of Biochemistry and Cancer Biology, Meharry Medical College, Nashville, Tennessee, USA; 3 Department of Molecular Pharmacology, Albert Einstein College of Medicine, Bronx, New York, USA; 4 Laboratory of Molecular Nutrition of the Institute for Personalized Medicine, Sechenov First Moscow State Medical University, Moscow, Russia

**Keywords:** EAAT2, GLT-1, excitotoxicity, manganese, neuron-restrictive silencing factor, neurotoxicity, RE1-silencing transcription factor, Parkinson's disease, astrocyte–neuron coculture, dopaminergic neurons, Δψm, mitochondrial membrane potential, AD, Alzheimer's disease, CBP, CREB-binding protein, cDNA, complementary DNA, ChIP, chromatin immunoprecipitation, co-IP, coimmunoprecipitation, COS7, CV-1 in Origin with SV40 cell line 7, CREB, cAMP response element–binding protein, DAPA, DNA affinity precipitation assay, DMEM, Dulbecco's modified Eagle's medium, DN-REST, dominant-negative form of REST, EAAT1, excitatory amino acid transporter 1, EAAT2, excitatory amino acid transporter 2, ENCODE, encyclopedia of DNA elements, EV, empty vector, GLAST, glutamate aspartate transporter 1, GLT-1, glutamate transporter 1, HDAC, histone deacetylase, HRP, horseradish peroxidase, IL, interleukin, LUHMES, Lund human mesencephalic, MEM, minimum essential media, Mn, manganese, MPTP, 1-methyl-4-phenyl-1,2,3,6-tetrahydropyridine, NRSE, neuron-restrictive silencer element, PD, Parkinson's disease, PLA, proximity ligation assay, PROMO, prediction of transcription factor binding sites, qPCR, quantitative PCR, RE1, repressor element 1, REST, repressor element 1-silencing transcription factor, ROS, reactive oxygen species, YY1, Yin Yang 1

## Abstract

Chronic exposure to high levels of manganese (Mn) leads to manganism, a neurological disorder with similar symptoms to those inherent to Parkinson's disease. However, the underlying mechanisms of this pathological condition have yet to be established. Since the human excitatory amino acid transporter 2 (EAAT2) (glutamate transporter 1 in rodents) is predominantly expressed in astrocytes and its dysregulation is involved in Mn-induced excitotoxic neuronal injury, characterization of the mechanisms that mediate the Mn-induced impairment in EAAT2 function is crucial for the development of novel therapeutics against Mn neurotoxicity. Repressor element 1-silencing transcription factor (REST) exerts protective effects in many neurodegenerative diseases. But the effects of REST on EAAT2 expression and ensuing neuroprotection are unknown. Given that the EAAT2 promoter contains REST binding sites, the present study investigated the role of REST in EAAT2 expression at the transcriptional level in astrocytes and Mn-induced neurotoxicity in an astrocyte–neuron coculture system. The results reveal that astrocytic REST positively regulates EAAT2 expression with the recruitment of an epigenetic modifier, cAMP response element-binding protein–binding protein/p300, to its consensus binding sites in the EAAT2 promoter. Moreover, astrocytic overexpression of REST attenuates Mn-induced reduction in EAAT2 expression, leading to attenuation of glutamate-induced neurotoxicity in the astrocyte–neuron coculture system. Our findings demonstrate that astrocytic REST plays a critical role in protection against Mn-induced neurotoxicity by attenuating Mn-induced EAAT2 repression and the ensuing excitotoxic dopaminergic neuronal injury. This indicates that astrocytic REST could be a potential molecular target for the treatment of Mn toxicity and other neurological disorders associated with EAAT2 dysregulation.

In the mammalian central nervous system, extracellular glutamate levels are tightly regulated to maintain normal physiological excitatory signals (1), preventing glutamate accumulation and ensuing excitotoxic neuronal injury (2, 3), which involves overstimulation of postsynaptic glutamate receptors, and downstream toxic processes (4), such as elevated calcium (Ca^2+^) influx, increased reactive oxygen species (ROS), and decreased ATP levels (for review, see Ref. (5)). Importantly, excitotoxic brain lesions are associated with several neurodegenerative disorders including Huntington's disease (6), amyotrophic lateral sclerosis (7, 8), stroke, traumatic brain injury (9, 10), Alzheimer's disease (AD) (11), Parkinson's disease (PD) (12), and manganism (13).

Chronic exposure to elevated levels of manganese (Mn) causes a neurological disorder referred to as manganism, sharing similar pathological changes to those of PD (14, 15, 16, 17, 18, 19). Although there have been extensive studies to understand Mn-induced neurotoxicity, the molecular mechanisms involved are yet to be established. Mn is known to induce oxidative stress, mitochondrial impairment, inflammation, and autophagy dysfunction (for review, see Ref. (20)). In addition, growing evidence indicates that glutamate dysregulation and excitotoxicity play a critical role in Mn-induced neurotoxicity (13, 21).

Excitotoxic neuronal injury associated with Mn-induced glutamate dysregulation in the brain is directly associated with impairment of astrocytic glutamate transporters, excitatory amino acid transporter 1 (EAAT1) and EAAT2 (or glutamate-aspartate transporter [GLAST] and glutamate transporter 1 [GLT-1], respectively, in rodents) (22), which are critical for the reuptake of synaptic glutamate, preventing glutamate accumulation and ensuing neuronal injury (for review, see Ref. (23)). Dysregulation of these astrocytic glutamate transporters is linked to multiple neurological diseases including AD, PD, amyotrophic lateral sclerosis, schizophrenia, epilepsy, ischemia, and autism (for review, see Ref. (23)). We have also reported that Mn reduced GLAST (EAAT1) and GLT-1 (EAAT2) at the transcriptional level in astrocytes (24, 25) and the mouse brain (26, 27). Although GLAST plays a major role in several brain regions such as cerebellum, GLT-1 (EAAT2) is the primary astrocytic glutamate transporter, accounting for over 90% of synaptic glutamate clearance (4, 28). Its impairment is closely linked to various neurological disorders (28, 29). Thus, understanding the regulatory mechanisms of EAAT2 expression is critical for astrocytic function and neuronal survival.

Transcriptional regulatory mechanisms of EAAT2 expression have been studied to a great extent (for review, see Ref. (23)). Several transcription factors, including cAMP response element–binding protein (CREB), NF-κB, and Yin Yang 1 (YY1), regulate EAAT2 expression. Mn activated YY1 expression, which in turn repressed EAAT2 expression by binding to YY1 consensus sites in the EAAT2 promoter, collaborating with histone deacetylases (HDACs). Interestingly, YY1 directly regulates neuron-restrictive silencing transcription factor/repressor element 1 (RE1)-silencing transcription factor (REST) in SH-SY5Y cells (30), suggesting the potential role of REST in Mn-induced neurotoxicity *via* YY1.

REST regulates genes involved in neurogenesis, differentiation, axonal growth, vesicular transport, and glutamate signaling (31, 32, 33, 34, 35, 36). Dysregulation of REST is implicated in several neurodegenerative disorders, including AD, PD, Huntington's disease, and ischemia (37, 38, 39, 40). REST was initially described as a transcriptional repressor as it repressed many neuronal genes in non-neuronal cells by binding to its *cis*-element known as RE1 (also known as neuron-restrictive silencer element [NRSE]) (41, 42) and collaborating with gene expression modifiers, such as HDAC, mSin3a, and CoREST (43, 44). However, recent studies have shown that REST regulates at least 2000 target genes (45, 46), exerting both repression and activation (47, 48, 49). Particularly, REST activates several genes related to neuroprotective effects including tyrosine hydroxylase, B-cell lymphoma 2, nuclear factor erythroid 2–related factor 2, and catalase (38, 50). These findings indicate that REST can repress or activate its target gene expression in a cellular context- and microenvironment-dependent manner (45, 51).

Growing evidence has shown that REST exerts neuroprotective effects against several neurodegenerative diseases, including AD (38), PD (39) and experimental 1-methyl-4-phenyl-1,2,3,6-tetrahydropyridine (MPTP)–induced PD models (52). Reduced REST levels exacerbated mitochondrial dysfunction and correlated with α-synuclein accumulation in an α-synuclein-overexpressed PD mouse model (53). Mn decreased REST expression in dopaminergic neuronal cells, leading to a decrease in tyrosine hydroxylase expression, resulting in neuronal toxicity (50). REST increased expression of genes associated with antioxidant, antiapoptotic, and anti-inflammatory functions (38, 50). REST deficiency increased neuronal excitability and cell death in *Caenorhabditis elegans*, indicating the role of REST in preventing excitotoxic neuronal injury (54).

While mechanisms of neuroprotective effects of REST in neurons have been studied to a great extent, the role of astrocytic REST in neuroprotection has yet to be fully appreciated. Astrocytic REST has been shown to regulate general glial functions, such as glia reprogramming (55), differentiation (56, 57), and dense core vesicle formation (58). REST also modulates genes related to mitochondrial function, inflammation, and synaptic plasticity in astrocytes (56). Notably, astrocytic REST has been reported to play a critical role in attenuating inflammatory responses and neurotoxicity where its deletion exacerbated the MPTP-induced motor deficits and dopaminergic neurotoxicity in mice (59). These findings indicate that astrocytic REST may play a crucial role in neuroprotection, requiring further investigation to understand its molecular mechanisms.

In this study, we investigated the ability of REST to increase EAAT2 (GLT-1) expression and function in astrocytes, leading to neuroprotection against Mn-induced dopaminergic toxicity, which is relevant to manganism, a PD-like neurological disorder, by attenuating Mn-reduced glutamate uptake and thus, preventing excitotoxicity. Our findings demonstrate that REST increased EAAT2 expression by binding to two RE1-binding motifs identified for the first time in the EAAT2 promoter region, and attenuated Mn-induced EAAT2 repression. In addition, astrocytic REST attenuated Mn-induced excitotoxic dopaminergic neuronal injury by increasing astrocytic EAAT2 function, resulting in decreased extracellular glutamate accumulation in an astrocyte–neuron coculture system. Combined, these findings indicate that astrocytic REST exerts neuroprotective effects against Mn neurotoxicity, at least in part by upregulation of EAAT2 expression and glutamate uptake in astrocytes.

## Results

### REST positively regulates EAAT2 expression at the transcriptional level in astrocytes

**Figure 1:**
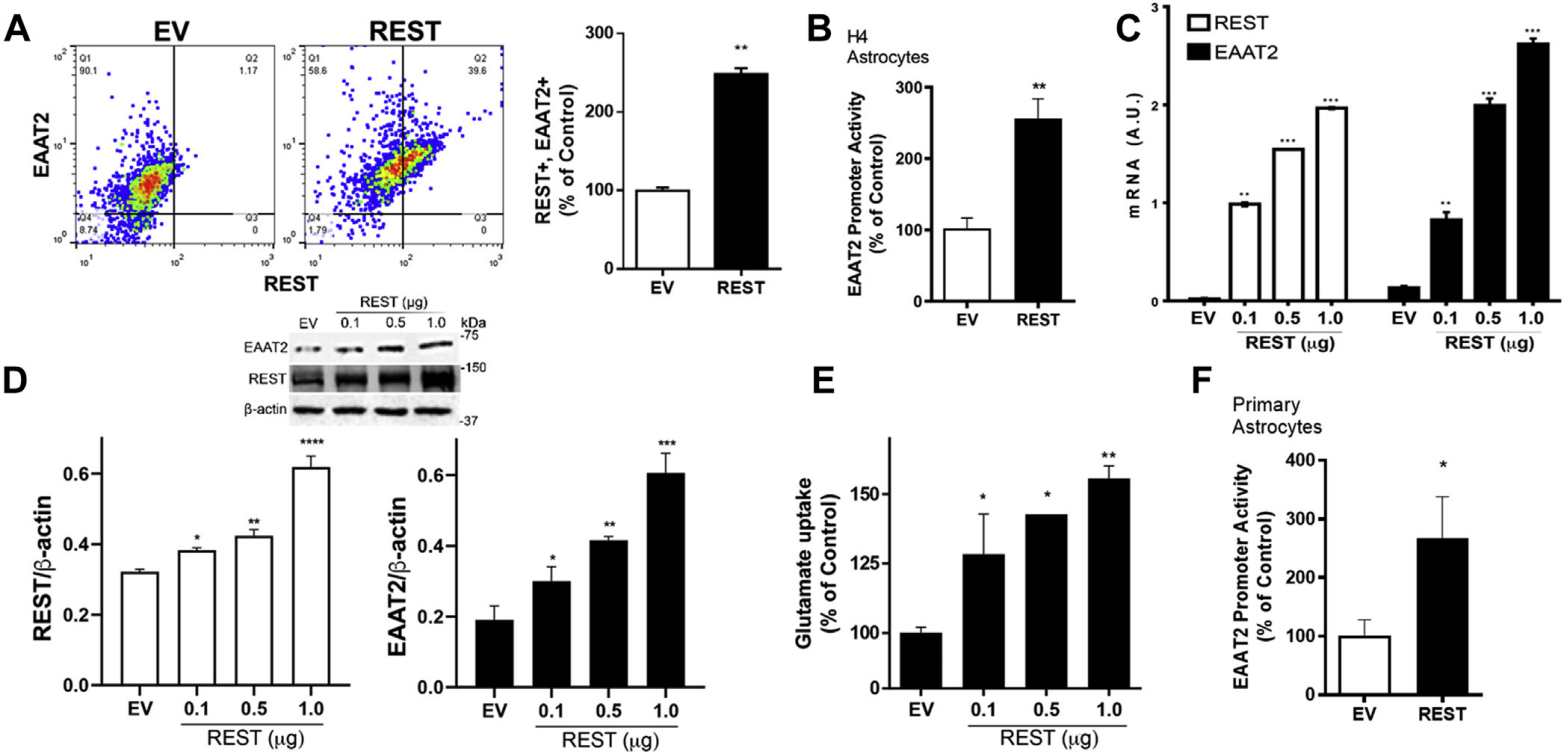
**REST positively regulates EAAT2 expression at the transcriptional level.***A*, after REST (1 μg) or empty vector (EV) transfection, H4 astrocytes were immunostained with anti-EAAT2 and anti-REST antibodies, followed by flow cytometry as described in the Experimental procedures section with quantification. *B*, astrocytes were cotransfected with the EAAT2 promoter and REST expression vectors, followed by luciferase assay as described in the Experimental procedures section. *C* and *D*, after REST transfection, astrocytes were prepared to measure levels of mRNA and protein of EAAT2 and REST, followed by quantitative PCR (*C*) and Western blotting (*D*), respectively. *E*, after REST transfection, glutamate uptake was assessed by measuring intracellular glutamate levels. *F*, the effects of REST on EAAT2 promoter activity were determined in primary mouse astrocytes. ∗*p* < 0.05; ∗∗*p* < 0.01; ∗∗∗*p* < 0.001; ∗∗∗∗*p* < 0.0001 compared with the controls (Student's *t* test or one-way ANOVA followed by Tukey's post hoc test; n = 3 of biological replicates). Data are expressed as mean ± SD. The data shown are representative of three independent experiments. EAAT2, excitatory amino acid transporter 20; REST, repressor element 1-silencing transcription factor.

We first tested if REST regulated glutamate transporter EAAT2 expression in human H4 astrocytes and primary mouse astrocytes. The results showed that EAAT2-expressing cell numbers were positively correlated with REST-expressing cell numbers (Q2 in Fig. 1*A*), indicating that REST overexpression in H4 astrocytes significantly increased EAAT2 expression. REST overexpression significantly increased EAAT2 promoter activities, mRNA, and protein levels in human H4 astrocytes (Fig. 1, *B*–*D*), indicating that REST positively regulates EAAT2 expression at the transcriptional level. We also tested if REST-increased EAAT2 expression is functionally correlated with glutamate uptake by assessing intracellular glutamate levels in H4 astrocytes. REST overexpression increased glutamate uptake with a concomitant increase in EAAT2 expression (Fig. 1, *C*–*E*).

**Figure 2:**
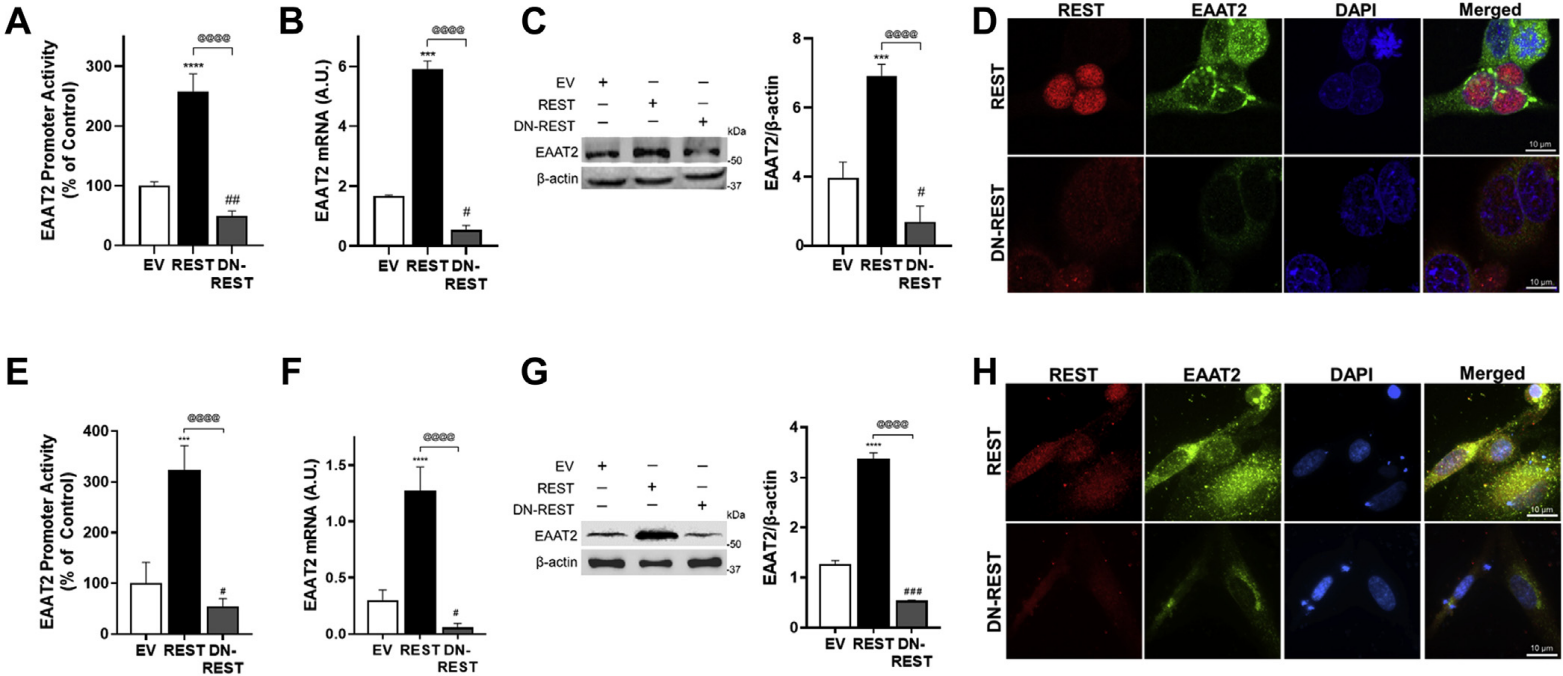
**Inhibition of REST function decreases EAAT2 expression.***A*–*H*, H4 astrocytes (*A*–*D*) and human primary astrocytes (*E*–*H*) were cotransfected with dominant-negative REST (DN-REST) and the EAAT2 promoter reporter vectors, followed by luciferase assay (*A* and *E*), quantitative PCR (*B* and *F*) and Western blotting (*C* and *G*). GAPDH and β-actin were used as loading controls of mRNA and protein, respectively. *D* and *H*, expression of EAAT2 and REST in astrocytes after overexpressed with REST or DN-REST vectors (×40 magnification with confocal microscope, scale represents 10 μm). ∗∗*p* < 0.01; ∗∗∗*p* < 0.001; ∗∗∗∗*p* < 0.0001; ^#^*p* < 0.05; ^##^*p* < 0.01; ^###^*p* < 0.001; compared with the controls; ^@@@@^*p* < 0.0001, compared with each other (one-way ANOVA followed by Tukey's post hoc test; n = 3). Data are expressed as mean ± SD. The data shown are representative of three independent experiments. EAAT2, excitatory amino acid transporter 20; REST, repressor element 1-silencing transcription factor.

Mouse and human primary astrocytes were also used to confirm the effects of REST on EAAT2, showing that REST increased EAAT2 expression (Figs. 1*F* and 2, *E*–*H*), indicating that REST is a positive regulator of EAAT2 transcription in astrocytes. To further confirm the role of REST in EAAT2 expression in astrocytes, we also used a dominant-negative form of REST (DN-REST), which expresses only its DNA-binding domain to compete with endogenous REST, thereby inhibiting nuclear translocation and function of endogenous REST (41). The results showed that DN-REST decreased EAAT2 promoter activity, mRNA, and protein levels in human H4 astrocytes (Fig. 2, *A*–*D*) as well as human primary astrocytes (Fig. 2, *E*–*H*). DN-REST inhibited the nuclear translocation of REST in human H4 and primary astrocytes (Fig. 2, *D* and *H*). These results confirm that REST is critical in the transcriptional activation of EAAT2 in astrocytes.

**Figure 3:**
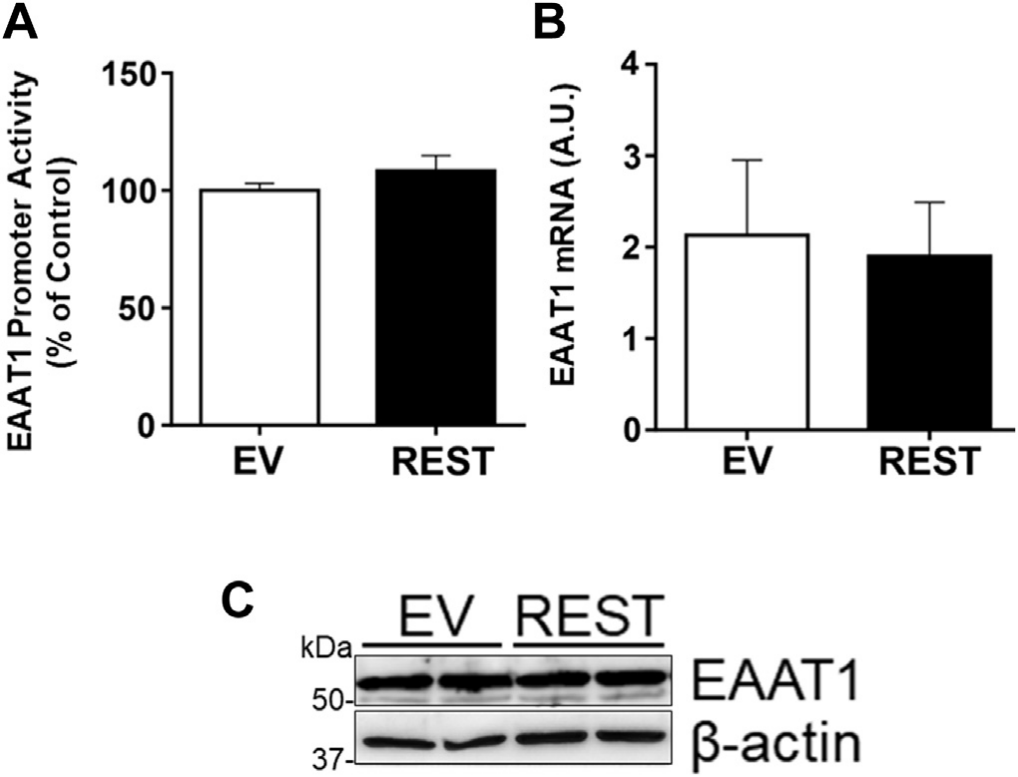
**REST does not alter EAAT1 expression in astrocytes.***A*–*C*, H4 astrocytes were cotransfected with the EAAT2 promoter reporter and/or REST expression vectors, followed by luciferase assay (*A*), quantitative PCR (*B*), and Western blotting (*C*). GAPDH and β-actin were used as loading controls of mRNA and protein, respectively. Data are expressed as mean ± SD. The data shown are representative of three independent experiments. EAAT2, excitatory amino acid transporter 20; REST, repressor element 1-silencing transcription factor.

On the other hand, REST did not affect astrocytic glutamate transporter EAAT1 promoter activities, mRNA, and protein levels (Fig. 3, *A*–*C*) in H4 astrocytes, indicating that REST is specific to EAAT2 regulation in astrocytes.

### REST binds its *cis*-regulatory sequences RE1 in the EAAT2 promoter

**Figure 4:**
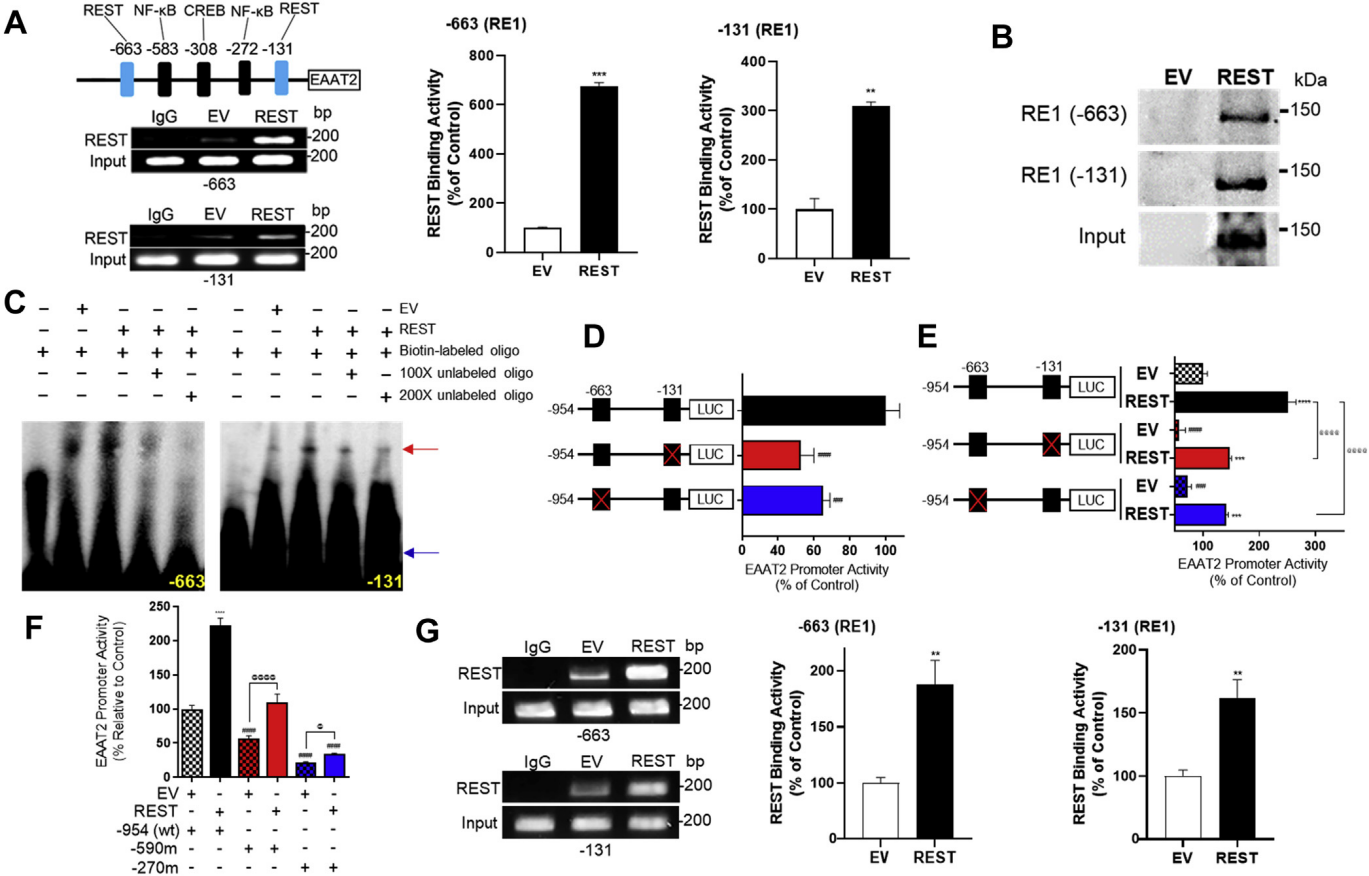
**Two RE1 consensus sites are critical for EAAT2 promoter activities and REST activation.***A*, two putative RE1 consensus sites (−663 and −131) in the EAAT2 promoter were identified. After transfection of REST, REST binding to its consensus sites in the EAAT2 promoter was determined by ChIP assay. Quantification of REST binding activities to these two RE1 consensus sites was measured by quantitative PCR from H4 astrocytes. *B*, DAPA was performed to determine REST binding to the oligonucleotide sequence of its consensus sites in astrocyte nuclear extracts after REST overexpression. *C*, EMSA was performed in nuclear extracts prepared from H4 astrocytes as described in the Experimental procedures section. The *red arrow* indicates the protein–DNA complex form for both RE1 consensus sites, and the *blue arrow* indicates unbound oligos. *D*, site-directed mutagenesis of two RE1 conserved motifs on EAAT2 promoter, followed by luciferase assay. *E*, after astrocytes were cotransfected with WT or mutant EAAT2 promoter and REST expression vectors, promoter activity was determined by luciferase assay. *F*, after H4 astrocytes were cotransfected with WT or deletion constructs of EAAT2 promoter and REST expression vectors, luciferase assay was performed. *G*, after transfection of REST in human primary astrocytes, REST binding to its consensus sites in the EAAT2 promoter was determined by ChIP assay, followed by quantitative PCR to quantify REST binding activities to these two RE1 consensus sites of the EAAT2 promoter. ∗∗*p* < 0.01; ∗∗∗*p* < 0.001; ∗∗∗∗*p* < 0.0001; ^#^*p* < 0.05; ^##^*p* < 0.01; ^###^*p* < 0.001; ^####^*p* < 0.0001 compared with the controls; ^@^*p* < 0.05; ^@@@@^*p* < 0.0001 compared with each other (one-way ANOVA followed by Tukey's post hoc test; n = 3). Data are expressed as mean ± SD. The data shown are representative of three independent experiments. ChIP, chromatin immunoprecipitation; DAPA, DNA affinity precipitation assay; EAAT2, excitatory amino acid transporter 2; RE1, repressor element 1; REST, repressor element 1-silencing transcription factor.

Since REST increased EAAT2 expression at the transcriptional level in astrocytes, we investigated if the EAAT2 promoter contains an NRSE (also known as RE1) where REST would putatively bind to regulate EAAT2 expression by a database search for the RE1 consensus sequences in the EAAT2 promoter. We analyzed the 954-bp sequences of the EAAT2 promoter region using the PROMO (prediction of transcription factor binding sites), ENCODE (encyclopedia of DNA elements), and JASPAR databases and identified that there are two putative 21-bp RE1 consensus sequences located in −663 and −131 upstream of the EAAT2 transcription initiation site (Fig. 4*A*) from the JASPAR 2020 (60) and confirmed by PROMO and ENCODE software. These two RE1 sites are independent of other previous positive regulators, such as NF-κB and CREB, to upregulate EAAT2 expression (61).

To determine if REST directly binds these putative binding sites, we performed a chromatin immunoprecipitation (ChIP) assay. The results showed that REST bound both of its *cis*-element sites in the EAAT2 promoter and increased its binding with higher REST expression in H4 astrocytes (Fig. 4*A*). The control group with empty vector (EV) transfection also showed low levels of REST binding for both sites in the EAAT2 promoter by endogenous REST protein. We also employed DNA affinity precipitation assay (DAPA) and EMSA to confirm that REST protein forms a DNA–protein complex with the REST-binding consequence sequences. As shown in Figure 4, *B* and *C*, oligonucleotides for both RE1 consensus sites of the EAAT2 promoter interacted with REST protein of ∼120 kDa, indicating that the full-length REST bound to these consensus sequence motifs (Fig. 4*B*). Interaction of these RE1 sites with REST protein was specific as excess of biotinyl-unlabeled RE1-specific oligonucleotides blocked the formation of this DNA–protein complex (Fig. 4*C*).

Inactivation of REST by mutations of the REST-binding sequences in the EAAT2 promoter also decreased EAAT2 promoter activities. Mutation of either of the two REST-binding sites reduced EAAT2 promoter activities (Fig. 4*D*), and REST overexpression increased EAAT2 promoter activities in both mutant constructs (Fig. 4*E*), indicating both of REST-binding sites are critical for activation of EAAT2 promoter activities. The enhancing effect of REST on deletion constructs of the EAAT2 promoter also supported these two RE1 sites in the EAAT2 promoter (Fig. 4*F*). Moreover, EAAT2 deletion mutants from −590 and −270 contained one RE1 *cis*-element (−131), but the effects of REST on EAAT2 promoter activation were higher in −590 than that of −270. This indicates that other factors including transcription factor CREB might contribute to this difference since the *cis*-element of CREB is located in the −308 site of the EAAT2 promoter, possibly collaborating with REST to enhance the EAAT2 promoter.

We have also determined if REST directly binds to these putative binding sites in primary human astrocytes by ChIP assay. Results showed that REST bound to both of its *cis*-element sites in EAAT2 promoter in primary human astrocytes (Fig. 4*G*).

### REST interacts with CBP/p300 and the transcription factor CREB for EAAT2 expression

**Figure 5:**
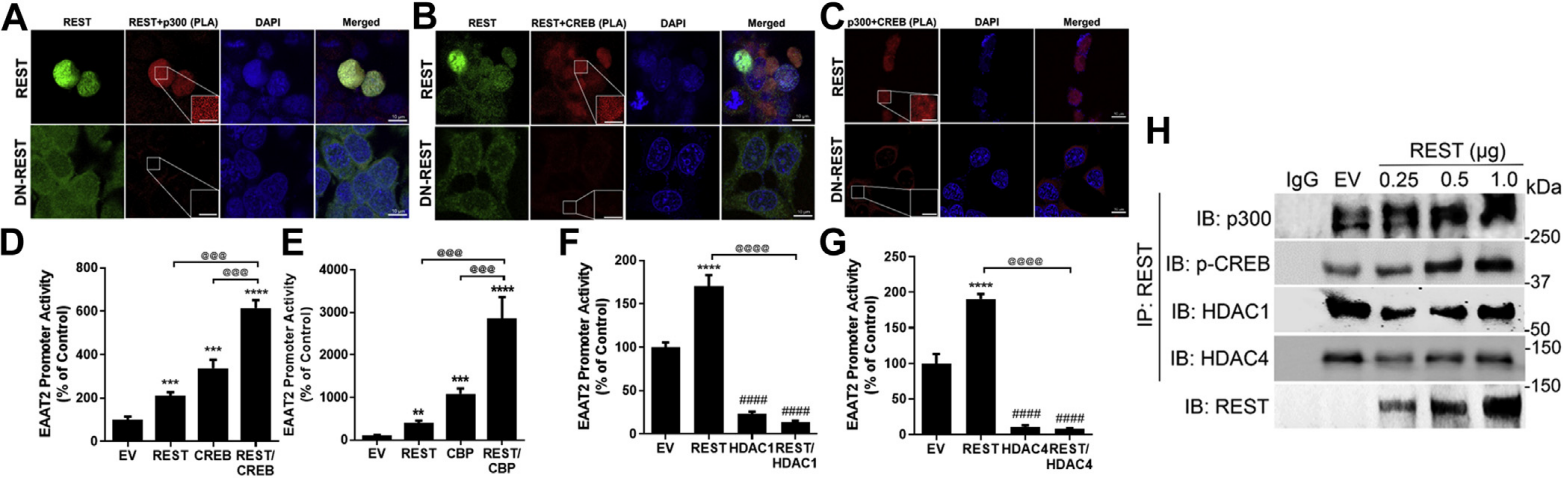
**REST interacts with CREB and CBP/p300 proximately to enhance EAAT2 transcription in astrocytes.***A*–*C*, REST directly interacts with CREB and CBP/p300 in astrocytes, showed by the proximity ligation assay (PLA) as described in the Experimental procedures section (×40 magnification with confocal microscope, the scale represents 10 μm). *Insets* show a higher magnification of the PLA puncta in the nuclear region (the scale represents 5 μm). *D*–*G*, H4 astrocytes were cotransfected with the EAAT2 promoter vector and REST with CREB (*D*), CBP/p300 (*E*), HDAC1 (*F*), or HDAC4 (*G*), followed by measurement of EAAT2 promoter activities by luciferase assay. *H*, H4 astrocytes were overexpressed with REST, followed by coimmunoprecipitation for CBP/p300, p-CREB, HDAC1, and HDAC4 in the nuclear extract. ∗∗*p* < 0.01; ∗∗∗*p* < 0.001; ∗∗∗∗*p* < 0.0001; ^####^*p* < 0.0001, compared with the controls; ^@@@^*p* < 0.001; ^@@@@^*p* < 0.0001, compared with each other (one-way ANOVA followed by Tukey's post hoc test; n = 3). Data are expressed as mean ± SD. The data shown are representative of three independent experiments. CBP, CREB-binding protein; CREB, cAMP response element–binding protein; EAAT2, excitatory amino acid transporter 2; HDAC, histone deacetylase.

We further explored the potential coactivators of REST including other transcription factors or epigenetic modifiers to regulate EAAT2 transcription (50, 61). We found that REST interacted with CREB, as well as histone acetyltransferases, such as CREB-binding protein (CBP) and p300, which are known to enhance EAAT2 transcription in the nuclear region by coimmunoprecipitation (co-IP) and proximity ligation assay (PLA) (Fig. 5, *A*, *B*, and *H*). The PLA assay was validated using several negative controls, including the primary antibodies for REST, CREB, and CBP/p300 in H4 astrocytes (Fig. S1). It appears that REST forms a larger multiplex by binding to the CREB and CBP/p300 complex for transcriptional activation of EAAT2 (61). The colocalization and complexing of REST with CBP/p300 and CREB in the nucleus were interfered by DN-REST, which blocks the nuclear translocation of REST from the cytosol in H4 astrocytes (Fig. 5*C*). Coexpression of REST with CREB or CBP/p300 further enhanced EAAT2 promoter activity in H4 astrocytes (Fig. 5, *D* and *E*). These results indicate that REST interacts with CREB and CBP/p300 to activate EAAT2 transcription.

Since REST is known to interact with HDAC proteins to repress its target genes' expression (32, 40, 62), we tested if HDACs modulate REST effects on EAAT2 promoter activity. HDAC1 from class I and HDAC4 from class II decreased EAAT2 promoter activities (Fig. 5, *F* and *G*) (24), and cotransfection of REST with either HDAC1 or HDAC4 abolished REST-induced EAAT2 promoter activity in H4 astrocytes (Fig. 5, *F* and *G*). However, REST appears to exhibit greater interaction with phosphorylated CREB (an active form of CREB) and CBP/p300 than with HDAC1 and HDAC4 (Fig. 5*H*), requiring further investigation to understand in depth the epigenetic regulation of EAAT2 by REST.

### REST attenuates Mn-induced repression of EAAT2 in astrocytes

**Figure 6:**
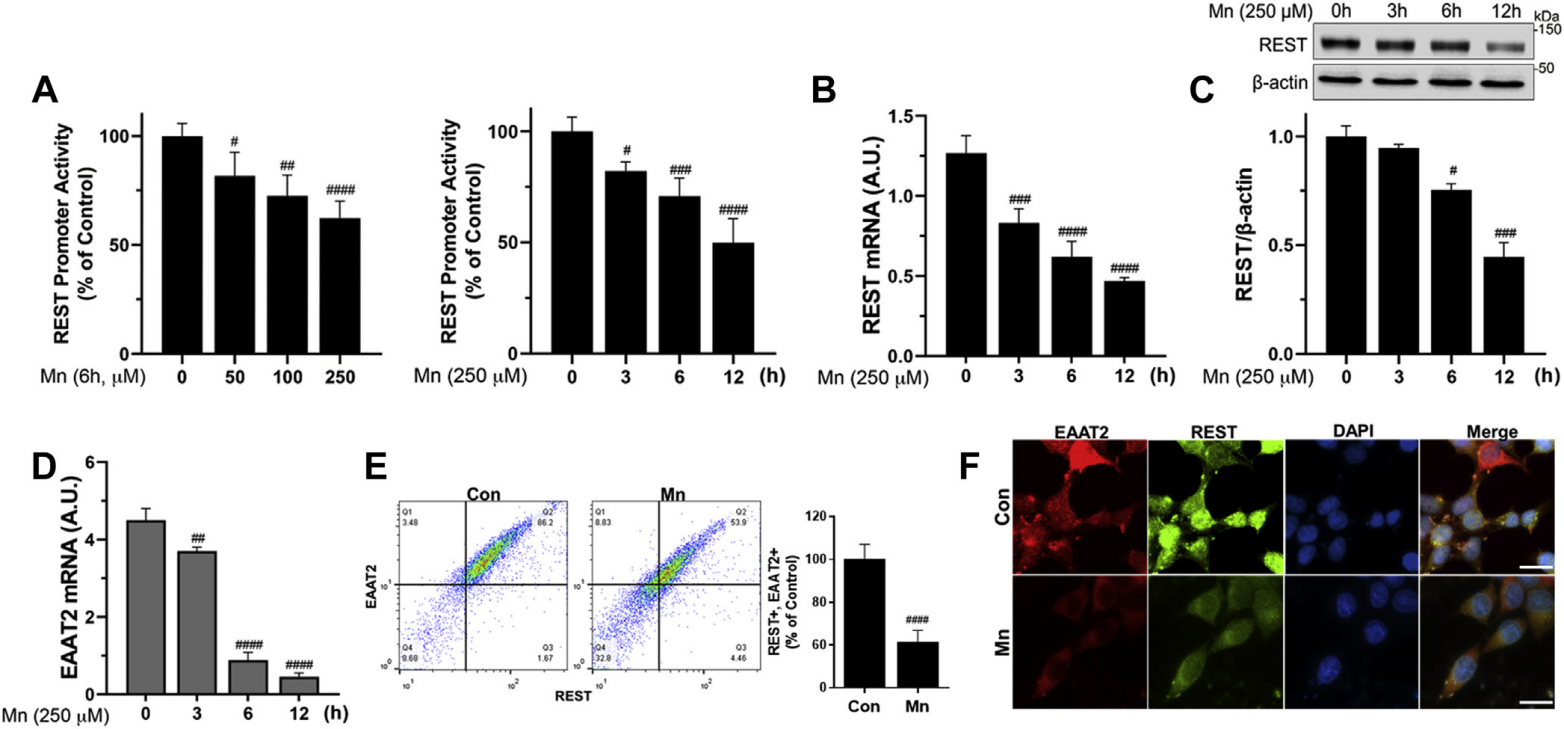
**Mn decreases REST expression at the transcriptional level in astrocytes.***A*, after transfection of REST promoter vector, H4 astrocytes were exposed to various concentrations and exposure times of Mn, followed by luciferase assay. *B*–*D*, after H4 astrocytes were exposed to Mn, REST mRNA (*B*) and protein (*C*) and EAAT2 mRNA (*D*) levels were assessed by quantitative PCR and Western blotting. *E* and *F*, after H4 astrocytes were exposed to Mn (250 μM) for 12 h, REST and EAAT2 protein expression was determined by flow cytometry (*E*) and immunocytochemistry (*F*) (×100 magnification with fluorescent microscope, the scale represents 10 μm). ^#^*p* < 0.05; ^##^*p* < 0.01; ^###^*p* < 0.001; ^####^*p* < 0.0001, compared with the controls (Student's *t* test or one-way ANOVA followed by Tukey's post hoc test; n = 3). Data are expressed as mean ± SD. The data shown are representative of three independent experiments. EAAT2, excitatory amino acid transporter 2; Mn, manganese; REST, repressor element 1-silencing transcription factor.

**Figure 7:**
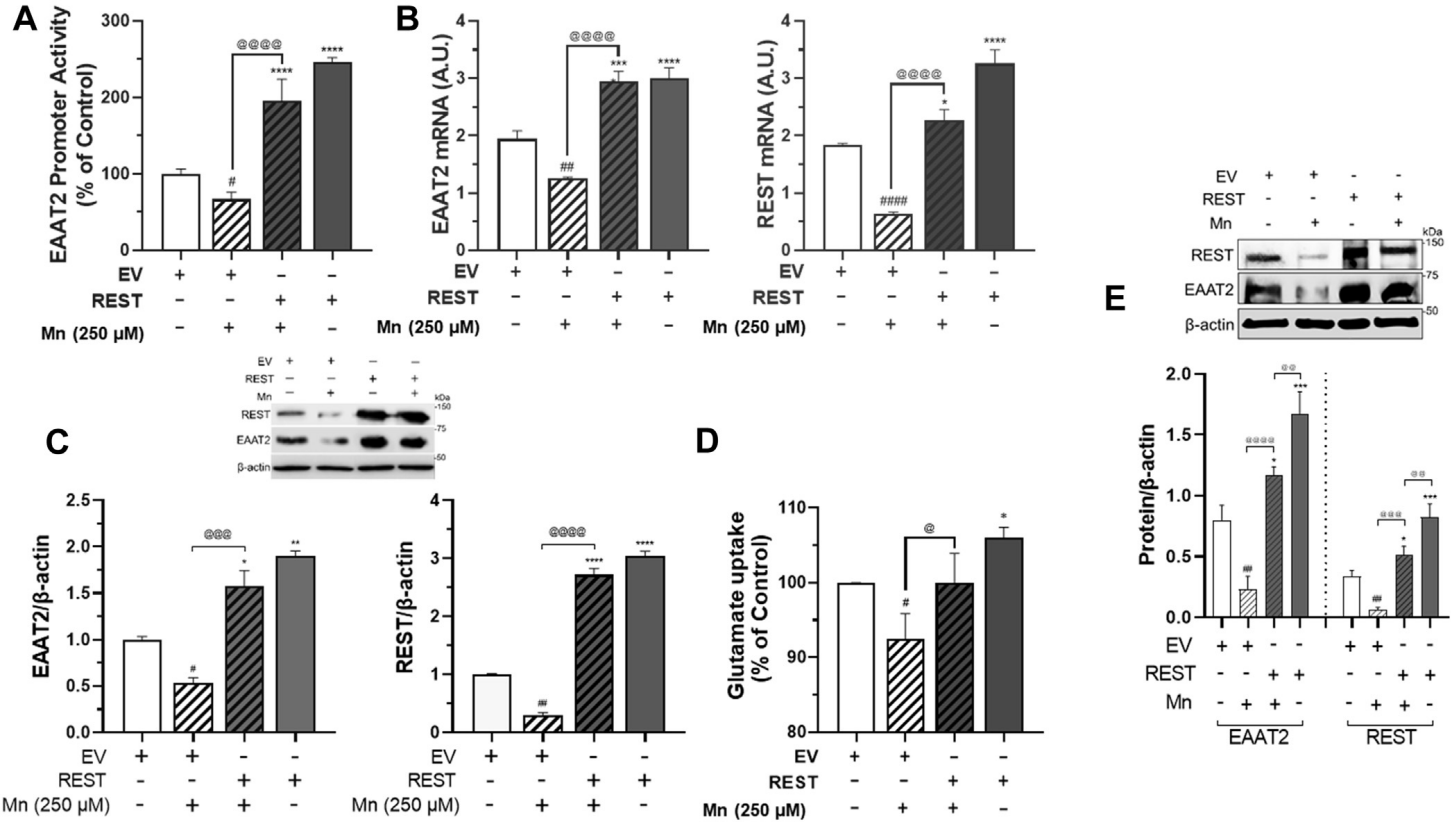
**REST attenuates Mn-repressed EAAT2 expression in astrocytes.***A*–*C*, after cotransfected with EAAT2 promoter and/or REST expression vectors, H4 astrocytes were exposed to Mn for 12 h to determine promoter activity, mRNA, and protein levels of EAAT2 and REST by luciferase assay (*A*), quantitative PCR (*B*), and Western blotting (*C*), respectively. *D*, REST-transfected astrocytes were exposed to Mn (250 μM) for 12 h, followed by intracellular glutamate assay for glutamate uptake as described in the Experimental procedures section. *E*, after REST was overexpressed in human primary astrocytes, cells were exposed to Mn (250 μM) for 12 h, followed by assessment of protein levels of REST and EAAT2 by Western blotting. ∗*p* < 0.05; ∗∗*p* < 0.01; ∗∗∗*p* < 0.001; ∗∗∗∗*p* < 0.0001; ^#^*p* < 0.05; ^##^*p* < 0.01; ^####^*p* < 0.0001, compared with the controls; ^@^*p* < 0.05; ^@@@^*p* < 0.001; ^@@@@^*p* < 0.0001 compared with each other (one-way ANOVA followed by Tukey's post hoc test; n = 3). Data are expressed as mean ± SD. The data shown are representative of three independent experiments. EAAT2, excitatory amino acid transporter 2; Mn, manganese; REST, repressor element 1-silencing transcription factor.

Since Mn decreases EAAT2 mRNA and protein levels *in vitro* as well as *in vivo* models (27, 63), and REST increases EAAT2 expression, we first tested whether Mn modulates REST at the transcriptional level along with the Mn-induced reduction in EAAT2 expression. The results showed that Mn decreased astrocytic REST promoter activities in a concentration- and time-dependent manner (Fig. 6*A*). Mn also decreased REST mRNA and protein levels (Fig. 6, *B* and *C*) with concomitant reduction of EAAT2 mRNA/protein levels (Fig. 6, *D* and *E*). Mn-induced decrease in EAAT2-expressing cells was comparably correlated with its decrease of REST-expressing H4 astrocytes (Q2 in Fig. 6*E*). Furthermore, Mn also decreased nuclear REST protein expression (Fig. 6*F*). To determine if REST attenuates Mn-reduced EAAT2 expression and function, EAAT2 expression and function in REST-overexpressing astrocytes were assessed. REST attenuated Mn-induced reduction of promoter activity, mRNA, and protein levels of EAAT2 (Fig. 7, *A*–*C*), as well as the Mn-induced reduction in glutamate uptake (Fig. 7*D*), indicating that REST, by enhancing EAAT2 expression, restores glutamate transport dysfunction in H4 astrocytes. We have also studied human primary astrocytes to determine if the effects of REST are analogous to those inherent to H4 astrocytes. Results showed that REST attenuated Mn-reduced EAAT2 protein levels in primary human astrocytes (Fig. 7*E*), confirming that REST protects against Mn-induced dysregulation of EAAT2 in normal astrocytes.

**Figure 8:**
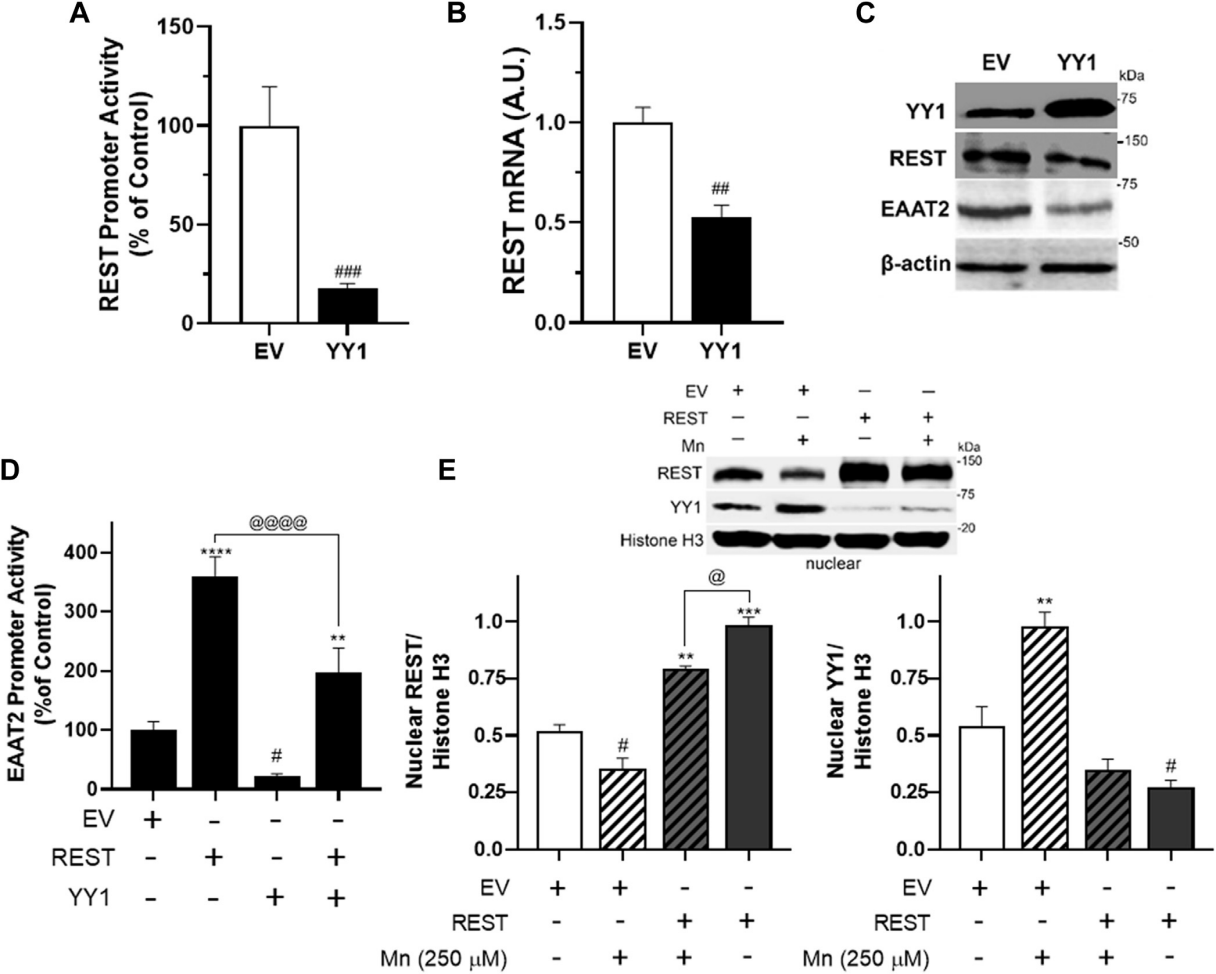
**YY1 represses REST, and REST overexpression abolished Mn-increased YY1 in astrocytes.***A*–*C*, H4 astrocytes were cotransfected with REST promoter and/or YY1 expression vectors, followed by assessing promoter activity, mRNA, and protein levels of REST, YY1, and/or EAAT2. *D*, after H4 astrocytes were cotransfected with EAAT2 promoter, REST, and/or YY1 vectors, EAAT2 promoter activity was measured. *E*, after REST-overexpressing H4 astrocytes were exposed to Mn, followed by nuclear fractionation, and Western blotting to determine nuclear REST and YY1 levels in H4 astrocytes. ∗∗*p* < 0.01; ∗∗∗*p* < 0.001; ∗∗∗∗*p* < 0.0001; ^#^*p* < 0.05; ^##^*p* < 0.01; ^###^*p* < 0.001 compared with the controls; ^@^*p* < 0.05; ^@@@@^*p* < 0.0001, compared with each other (Student's *t* test or one-way ANOVA followed by Tukey's post hoc test; n = 3). Data are expressed as mean ± SD. The data shown are representative of three independent experiments. EAAT2, excitatory amino acid transporter 2; Mn, manganese; REST, repressor element 1-silencing transcription factor; YY1, Yin Yang 1.

Since activation of YY1 is involved in Mn-decreased EAAT2 expression (24), a potential role of YY1 in Mn-decreased REST was assessed in astrocytes. The results showed that YY1 decreased REST promoter activities, mRNA, and protein levels (Fig. 8, *A*–*C*). In addition, REST overexpression attenuated YY1-induced reduction of EAAT2 promoter activity (Fig. 8*D*) and inhibited Mn-activated YY1 nuclear localization as well as Mn-reduced nuclear REST in H4 astrocytes (Fig. 8*E*). These findings indicate that the transcriptional repression of Mn of EAAT2 is highly dynamic, involving multiple mechanisms including direct YY1 binding to its binding motifs of the EAAT2 promoter region (24) and indirectly by YY1-induced reduction of REST.

### Astrocytic REST attenuates the excitotoxic dopaminergic neuronal injury of Mn in an astrocyte–neuron coculture

Studies have shown that Mn induced excitotoxic lesions in rat striatum (13) and dopaminergic neuronal injury in both *in vitro* and *in vivo* experimental models (26, 27, 64, 65). Mn-induced impairment of astrocytic glutamate transporters directly results in excess extracellular glutamate levels, leading to excitotoxic neuronal injury of adjacent neurons. Furthermore, it has been reported that dopaminergic neurons express glutamate receptors in mice and rhesus monkeys (66, 67). Thus, we tested if astrocytic REST attenuated Mn-induced excitotoxic dopaminergic neuronal injury because of the decrease of EAAT2 expression and function by Mn, using an astrocyte–neuron coculture system.

**Figure 9:**
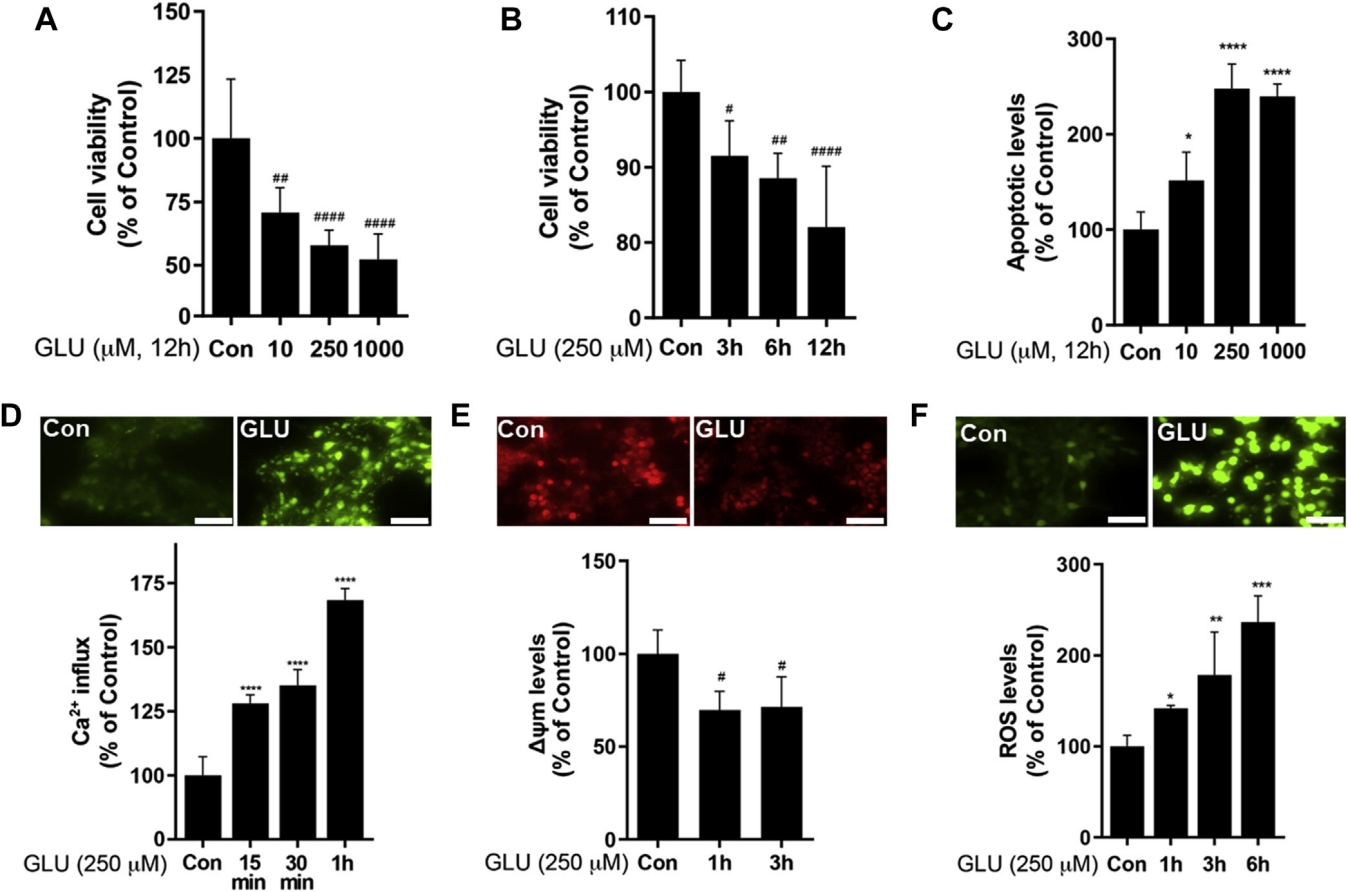
**Establishment of excitotoxic condition in dopaminergic cell–like LUHMES cell model.***A*–*C*, LUHMES cells were exposed to glutamate in a concentration- (*A*) and time-dependent (*B*) manner to determine excitotoxic neuronal injury by resazurin assay for cell viability and annexin V assay (*C*) for apoptosis as described in the Experimental procedures section. *D*–*F*, neuronal cells exposed to glutamate (250 μM, 12 h) were assessed for Ca^2+^ influx (*D*), mitochondrial membrane potential (Δψm) (*E*), and reactive oxygen species (ROS) (*F*) (×20 magnification with fluorescent microscope, the scale represents 50 μm). ∗*p* < 0.05; ∗∗*p* < 0.01; ∗∗∗*p* < 0.001; ∗∗∗∗*p* < 0.0001; ^#^*p* < 0.05; ^##^*p* < 0.01; ^####^*p* < 0.0001, compared with the controls (one-way ANOVA followed by Tukey's post hoc test; n = 6). Data are expressed as mean ± SD. The data shown are representative of three independent experiments. LUHMES, Lund human mesencephalic.

Neuronal excitotoxicity was assessed by measuring cell viability, apoptosis, Ca^2+^ influx/signaling, mitochondrial membrane potential (Δψm), and ROS levels in differentiated Lund human mesencephalic (LUHMES) cells as a dopaminergic cell–like model (Fig. 9). First, we established the optimal condition of glutamate exposure in LUHMES cells to induce excitotoxicity in an astrocyte–neuron coculture system. Glutamate induced cytotoxicity in neuronal cells with a decrease of cell viability and increased apoptotic levels in a concentration- and time-dependent manner (Fig. 9, *A*–*C*), leading to glutamate exposure at 250 μM for 12 h as an optimal condition to induce dopaminergic toxicity. Since overstimulation of glutamate receptors leads to an excessive influx of Ca^2+^, followed by impairment of Δψm, and ROS overproduction as early events leading to neuronal death, we measured Ca^2+^ influx up to 1 h, Δψm up to 3 h, and ROS up to 6 h. The results showed that glutamate induced the highest Ca^2+^ influx at 1 h in LUHMES cells (Fig. 9*D*), leading to subsequent dysregulation of several downstream processes, including mitochondrial function, oxidative stress, and apoptosis. Indeed, glutamate decreased Δψm (Fig. 9*E*) and increased ROS levels (Fig. 9*F*) in LUHMES cells.

**Figure 10:**
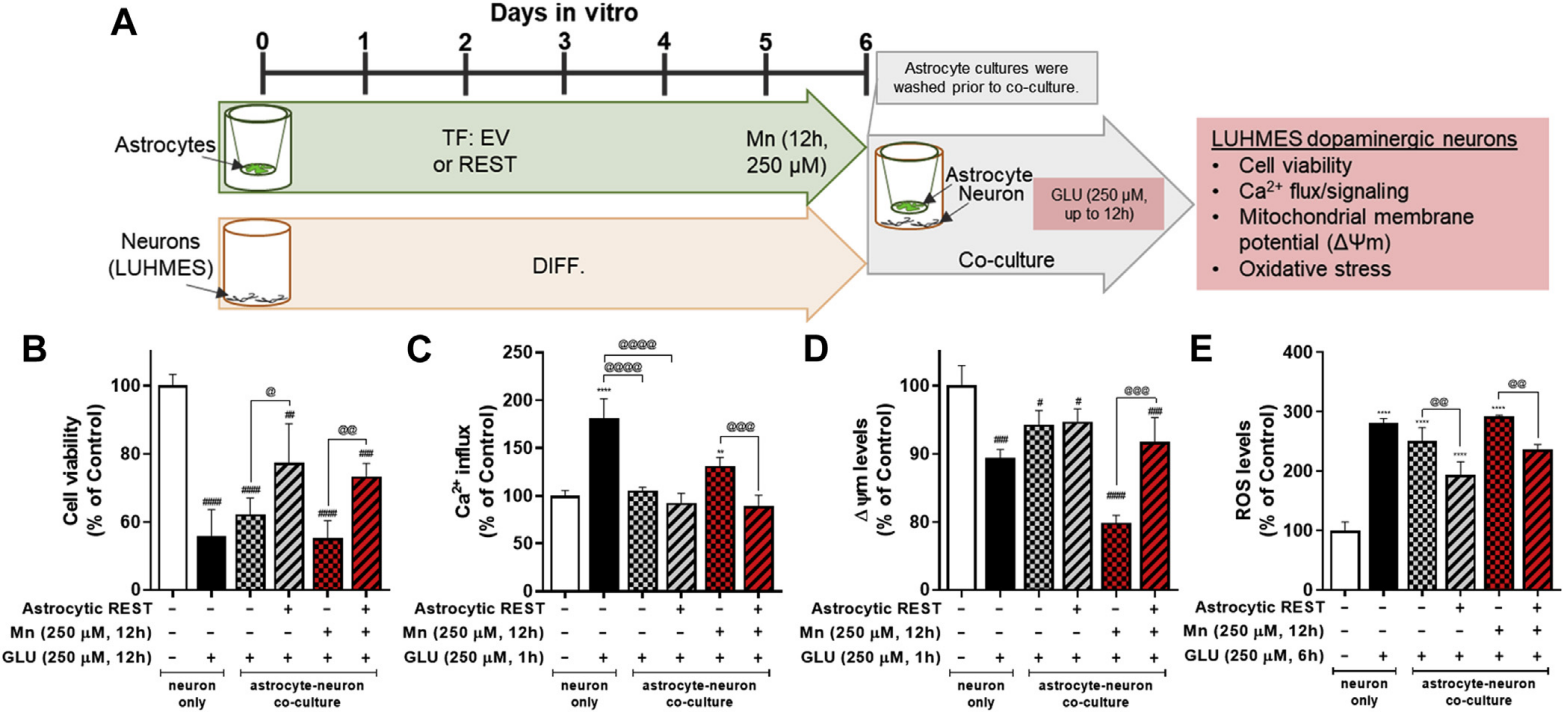
**Astrocytic REST attenuated Mn-induced excitotoxic neuronal injury in an astrocyte-LUHMES cell coculture.***A*, schematic representation of the experimental paradigm for H4 astrocyte-LUHMES coculture. *B*–*E*, effect of astrocytic REST in Mn-induced excitotoxic neuronal injury. REST-overexpressing astrocytes were exposed to Mn separately in transwells, followed by transferring transwells containing astrocytes into culture plates containing dopaminergic neuronal cells to create coculture conditions in the presence of glutamate (250 μM, 12 h). Neuronal toxicity was assessed for cell viability (*B*), Ca^2+^ influx (*C*), Δψm (*D*), and ROS production (*E*). ∗∗*p* < 0.01; ∗∗∗∗*p* < 0.0001; ^##^*p* < 0.01; ^###^*p* < 0.001; ^####^*p* < 0.0001, compared with the controls; ^@^*p* < 0.05; ^@@^*p* < 0.01; ^@@@^*p* < 0.001; ^@@@@^*p* < 0.0001 compared with each other (one-way ANOVA followed by Tukey's post hoc test; n = 6). Data are expressed as mean ± SD. The data shown are representative of three independent experiments. LUHMES, Lund human mesencephalic; Mn, manganese; REST, repressor element 1-silencing transcription factor; ROS, reactive oxygen species.

In order to determine the role of astrocytic REST in Mn-induced excitotoxic neuronal injury by impairing astrocytic EAAT2, REST-overexpressing H4 astrocytes were exposed to Mn (250 μM, 12 h) in transwell cultures. After removal of the Mn-containing culture media, transwells with astrocytes were placed on top of differentiated LUHMES cells that were cultured in 24-well plates, followed by exposure to glutamate (250 μM) for 12 h in the astrocyte–neuron coculture system (Fig. 10*A*). The results showed that REST-overexpressing astrocytes attenuated Mn effects on excitotoxic neuronal injury compared with EV (no REST)-expressing H4 astrocytes, as shown that it ameliorated glutamate toxicity on cell viability (Fig. 10*B*), Ca^2+^ influx (Fig. 10*C*), Δψm (Fig. 10*D*), and ROS levels (Fig. 10*E*) in LUHMES cells. This indicates that astrocytic REST attenuates Mn-induced excitotoxic neuronal injury.

## Discussion

Our findings demonstrate for the first time that REST/NRSF activates and enhances transcription of the astrocytic glutamate transporter EAAT2 (GLT-1) in human and mouse astrocytes by binding to two RE1/NRSE-binding consensus sites (−663 and −131) in the EAAT2 promoter region with the recruitment of CREB and CBP/p300, resulting in increased EAAT2 promoter activity, mRNA, and protein levels. Moreover, REST attenuates the repression of EAAT2 by Mn and the excitotoxic dopaminergic neuronal injury of Mn in an astrocyte–neuron coculture system. These results indicate that astrocytic REST plays a critical role in protective effects against Mn-induced excitotoxic neuronal injury, at least in part, by upregulation of the astrocyte-specific glutamate transporter EAAT2.

Since EAAT2 is essential in neuroprotection against excitotoxic neuronal injury, understanding mechanisms involved in regulation of EAAT2 are crucial. Our findings are the first to report that REST directly binds its two critical *cis*-regulatory elements (−663 and −131) in the EAAT2 promoter. Although REST is known mainly as a repressor, the results from the present study show that REST activates EAAT2 expression, supported by multiple experimental data with various approaches (Figs. 1 and 2). Several previous studies have reported that REST acts as a repressor to downregulate its target genes (68), but several recent studies have shown that REST activates its target genes (47, 48, 49), involved in neuroprotective mechanisms (38, 50). Studies have shown that one of the REST isoforms, REST4, which is truncated in the C-terminal domain of full-length REST, enhances gene expression either by directly binding to its *cis*-element sites, leading to transcriptional activation in COS7 (CV-1 in Origin with SV40 cell line 7) cells (69), or blocking its full-length REST counterpart that represses target genes by competing in PC12 (pheochromocytoma cell line 12) cells (70). Notably, we show that full-length REST (∼120 kDa) binds and activates the EAAT2 promoter, resulting in increased EAAT2 expression in astrocytes.

Several transcription factors, including NF-κB and CREB, modulate EAAT2 transcription (61). In particular, CREB positively regulates EAAT2 expression with at least one CREB-response element at −308 position of the EAAT2 promoter (Fig. 4*A*). Inactivation of this CREB-response element by mutation decreased EAAT2 expression (61). Several pharmacological agents, such as tamoxifen and raloxifene, enhance EAAT2 promoter activity *via* CREB activation in astrocytes (61). Studies suggest a strong link between REST and CREB in neuronal (50) and lung cancer cells (71), but no studies have previously shown that CREB activation plays a role in the upregulation of EAAT2 by REST. Our finding reveals that CREB cooperates with REST in enhancing EAAT2 expression (Fig. 5, *A*, *D*, and *H*) and attenuating Mn-induced reduction in EAAT2 expression and glutamate uptake. Previous studies also reported that REST and CREB are collaborating to exert neuroprotection as elevated levels of REST were correlated with CREB activation in the hippocampus, resulting in improved mood and behavior in epileptic patients (72).

Epigenetic modifiers modulate transcription factor regulation of target genes. Our novel findings showed that REST exhibited a more pronounced interaction with CBP/p300 over HDAC to enhance EAAT2 expression (61), forming a complex with CREB, suggesting that the roles of HDAC or CBP/p300 in modulation of REST effect on EAAT2 expression are context dependent and microenvironment dependent. Several transcription factors or pharmacological agents modulate both EAAT2 and EAAT1, the latter being another important astrocytic glutamate transporter, in a similar manner (for review, see Ref. (23)), but REST appears to upregulate EAAT2 selectively, supported by the results that REST does not alter EAAT1 transcription (Fig. 3).

EAAT2 dysregulation is observed in multiple neurological disorders including PD (73, 74) and manganism (21, 27, 50, 75). Studies have corroborated Mn-induced excitotoxic lesions in the striatum of rat brain (13) and increased glutamate levels in rat striatum (76). Our findings reveal that Mn-induced impairment in EAAT2 expression and function is closely associated with excitotoxic dopaminergic neuronal injury (Fig. 10), corroborating the propensity of Mn to decrease GLT-1 mRNA and protein levels in murine substantia nigra and striatal regions with dopaminergic injury (24, 63). It is of note that there is contradictory and inconclusive understanding of the neurotoxicity of Mn in relation to dopaminergic neuronal death. It has been reported that Mn-induced parkinsonism is distinct from idiopathic PD, where dopaminergic neurons of the substantia nigra pars compacta are specifically damaged, but Mn-induced parkinsonism is distinguishable from PD by the lack of therapeutic response of motor function to levodopa (19, 77). However, many studies have shown that Mn caused dopaminergic neuronal death in various experimental models (15, 26, 27, 50, 63, 65). These controversial findings of the effects of Mn on dopaminergic neurons in the substantia nigra could be due to different experimental settings and Mn exposure levels, requiring further studies.

YY1 plays a critical role in the Mn-induced downregulation of EAAT2 expression in astrocytes, as Mn-induced YY1 activation and expression lead to enhanced YY1 binding to its consensus sites as a repressor on the EAAT2 promoter region (+32 site in the EAAT2 promoter), resulting in repression of EAAT2 expression and impairment of glutamate uptake (24). Since REST enhances EAAT2 expression and attenuates Mn-induced repression of EAAT2, it is of interest to determine whether REST modulates the Mn–YY1–EAAT2 axis. Our findings reveal that Mn decreases REST expression at the transcriptional levels, and YY1 also represses REST transcription, as it decreases REST promoter activity, mRNA, and protein levels in astrocytes (Figs. 6 and 8). The underlying mechanism of YY1-induced repression of REST remains to be established. Notably, YY1 may regulate REST expression by directly binding the REST promoter region as there is a YY1 binding site in the REST promoter (30). Yet, YY1 positively regulates REST expression in SH-SY5Y neuronal cells, indicating that YY1 regulates REST in a cell type- and context-dependent manner. Moreover, Mn-induced repression of EAAT2 may be bifunctional, namely (1), directly by activating YY1, which represses EAAT2 by binding to its consensus site in the EAAT2 promoter region (+32) (24) or (2) indirectly by repressing REST.

Growing evidence shows that REST is neuroprotective, and its dysfunction may contribute to the neuropathology of multiple neurological disorders, such as PD, dementia with Lewy body (39, 53), and AD (38). Decreased REST levels were correlated with higher oxidative stress and apoptotic events with dementia in AD brains compared with normal healthy brains (38). REST was sequestrated in Lewy bodies and cytosol, resulting in the absence or low REST levels in the nucleus of PD and dementia with Lewy body patients (39). Although the protective mechanisms of REST have yet to be fully understood, it has been shown that REST enhances antioxidant, anti-inflammatory, and antiapoptotic effects (38, 50, 59). It has been reported that astrocytic REST deletion exacerbated MPTP-induced activation of astrocytes and microglia, and dopaminergic neurotoxicity in mice, at least in part by further increased levels of proinflammatory cytokines, including interleukin-1β (IL-1β), IL-6, cyclooxygenase 2, and upregulating stress-related inducible nitric oxide synthase (59). REST is also known to exert neuroprotection by inhibiting excessive glutamatergic and cholinergic activation in aging brain, promoting neuronal survival and longevity (54). In support of this report, our findings show that astrocytic REST protects against Mn-induced excitotoxic neuronal injury by attenuating the Mn-induced decrease in EAAT2 expression and glutamate uptake in an astrocyte–neuron coculture system (Fig. 10). These results corroborate previous studies establishing that EAAT2 overexpression in astrocytes rescues motor neurons exposed to high levels of glutamate (78).

**Figure 11:**
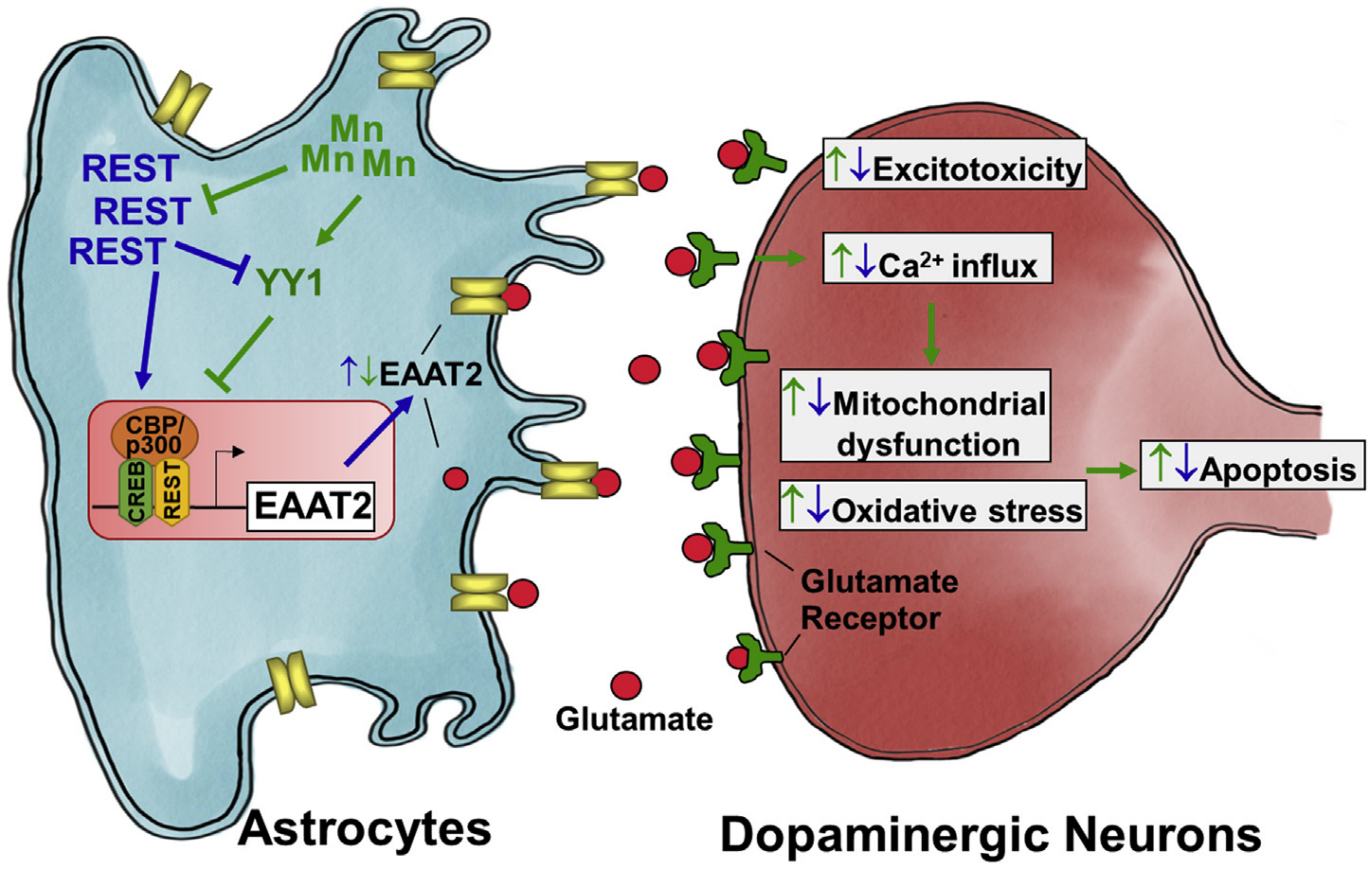
**Schematic diagram of the proposed mechanism for the role of REST in Mn-induced EAAT2 dysregulation in astrocytes and excitotoxicity.** Mn-induced reduction of REST in astrocytes decreases levels of glutamate transporter EAAT2 in the plasma membranes, resulting in extracellular glutamate accumulation leading to dopaminergic toxicity (*green arrows*). REST also attenuates Mn-induced YY1 activation and EAAT2 repression in H4 astrocytes (*blue arrows*). These attenuations of REST of Mn-reduced EAAT2 expression and glutamate uptake protect dopaminergic neurons against excitotoxic damage. The findings suggest that the astrocytic REST can be a critical target for neuroprotection against Mn-induced dopaminergic toxicity. EAAT2, excitatory amino acid transporter 20; Mn, manganese; REST, repressor element 1-silencing transcription factor; YY1, Ying Yang 1.

Taken together, our findings demonstrate for the first time that REST is a positive regulator of EAAT2 (GLT-1), binding directly to two sites of newly identified REST-binding consensus sequences in the EAAT2 promoter, forming a complex with CBP/p300 and CREB. Furthermore, astrocytic REST attenuated Mn-induced reduction of EAAT2 mRNA/protein levels and excitotoxic dopaminergic neurotoxicity in an astrocyte–neuron coculture, demonstrating the neuroprotective role of astrocytic REST in Mn-induced excitotoxic dopaminergic neuronal injury (Fig. 11). Although we used a human dopaminergic neuronal cell line to determine astrocytic REST effects, any other neuronal types that are influenced by astrocytic glutamate transporters are also likely protected by astrocytic REST. These indicate that astrocytic REST could be a critical molecular target for developing novel neurotherapeutics against excitotoxic neuronal injury, which is also inherent to many other neurological disorders.

## Experimental procedures

### Materials

Manganese chloride (MnCl_2_), dimethyl sulfoxide, and resazurin were purchased from MilliporeSigma. All cell culture media, including trypsin–EDTA, minimum essential media (MEM), Dulbecco's modified Eagle's medium (DMEM), DMEM/F-12, and Opti-MEM, were obtained from Gibco. The chloromethyl derivative of 2′,7′-dichlorodihydrofluorescein diacetate, an ROS molecular probe, annexin V, an apoptosis probe, Fluo-4AM, an intracellular calcium fluorescence probe, and tetramethylrhodamine ethyl ester, a mitochondrial membrane potential probe, were purchased from Invitrogen. Antibodies for REST (07-579) and ChIP-validated REST (17-10456) were acquired from MilliporeSigma. Antibodies for EAAT2 (sc-365634), REST (sc-374611), YY1 (sc-7341), CBP/p300 (sc-32244), phosphorylated CREB (sc-81486), CREB (sc-186), HDAC1 (sc-81598), and β-actin (sc-47778) were obtained from Santa Cruz Biotechnology. Antibodies for HDAC4 (2072) were obtained from Cell Signaling Technology. Antibodies for EAAT2/GLT-1 (ab41621), horseradish peroxidase (HRP)–conjugated rabbit antimouse IgG (ab6728), HRP-conjugated goat anti-rabbit IgG (ab97051), and goat anti-rabbit, antimouse antichicken antibodies conjugated with Alexa Fluor 488 or Alexa Fluor 568 were obtained from Abcam. All chemicals were prepared in PBS, double-distilled water or dimethyl sulfoxide, and diluted to working concentrations in Opti-MEM prior to use. Expression vectors REST-myc and its empty control vector (pCMV6-entry) were from OriGene Technologies. Control and expression vectors for CBP/p300, CREB, HDAC1, HDAC4, YY1-FLAG, pHR′-NRSF-eGFP, and TetO-FUW-DN-REST were obtained from Addgene. The human EAAT2 promoter vector was from Albert Baldwin (University of North Carolina at Chapel Hill). Human EAAT1 promoter was from David J. Volsky (Columbia University). The REST promoter plasmid was a gift from Dr Yvon Trottier (INSERM, France).

### Cell culture

Primary cultures of human and mouse astrocytes and a human H4 astrocyte cell line were used in the study. Human H4 (HTB-148) cells were obtained from American Type Culture Collection. Human primary astrocytes (HA; #1800) were purchased and prepared according to the manufacturer's recommendation (ScienCell Research Laboratories). To prepare primary mouse astrocytes, following careful removal of meninges, the cerebral hemispheres of 1-day-old newborn mice (C57BL6/J) were dissociated using Dispase II (Boehringer–Mannheim Biochemicals; neutral protease, Dispase Grade II). Twenty-four hours after the initial plating, the media were changed to preserve the adhering astrocytes and to remove neurons and oligodendrocytes. The cultures were maintained in MEM supplemented with 10% fetal bovine serum, 100 units/ml of penicillin, and 100 μg/ml of streptomycin. These cultures showed >95% positive staining for glial fibrillary acidic protein, an astrocyte-specific marker. All the experiments were performed 3 weeks after isolation. Human H4 astrocytes were maintained in DMEM supplemented with Gibco, 10% fetal bovine serum, 100 U/ml of penicillin, and 100 μg/ml of streptomycin. LUHMES cells were maintained in DMEM/F-12 with 1% N2 supplement (Gibco) and 40 ng/ml basic fibroblast growth factor (PeproTech). LUHMES cells were subcultured on culture flasks precoated with 50 μg/ml poly-l-ornithine and fibronectin. LUHMES cells were differentiated into morphologically and biochemically mature dopamine-like neurons following exposure to tetracycline, glial cell–derived neurotrophic factor, and dibutyryl-cAMP (PeproTech).

### Measurement of promoter activity

Astrocyte cultures were transfected with the human REST, EAAT2, or EAAT1 promoter plasmids using Lipofectamine 3000 or by electroporation as described previously (50). The mutations on RE1 consensus binding sites (−663 and −131 positions) in the human EAAT2 promoter plasmid were performed in this experiment. Moreover, REST, DN-REST, and YY1 plasmid vectors were cotransfected with the appropriate promoter plasmid vectors in astrocytes. After overnight transfections, the effects of various compounds on promoter activities were determined with the Bright-Glo luciferase assay kit (Promega) according to the manufacturer's instructions. We used deletion constructs (−590 and −270) generated previously (61) and RE1-mutated sites of the EAAT2 promoter in this study.

### Quantitative RT–PCR analysis

After astrocyte cultures were treated, total RNA was extracted from samples using the RNeasy Mini Kit (Qiagen), and 2 μg of purified RNA was transcribed to complementary DNA (cDNA) with a high-capacity cDNA reverse transcription kit (Applied Biosystems). Real-time quantitative PCR (qPCR) was performed using the CFX96 real-time PCR detection system (Bio-Rad). The reaction mixture contained 1 μl of each cDNA template, 0.4 μM of primers, and iQ SYBR Green Supermix (Bio-Rad). The total reaction volume was 25 μl. The following primers were used: human REST 5′-GTG AGC GAG TAT CAC TGG AGG-3′ (forward) and 5′-CCC ATT GTG AAC CTG TCT TGC-3′ (reverse); human EAAT2 5′-CAA CAG AGC CCT CTC TGA ATA C-3′ (forward) and 5′-GTA GGG TGG ATG GGA TAC AAT G-3′ (reverse); human EAAT1 5′-GGA GCA AAA CAA AGC CAG CA-3′ (forward) and 5′-ATT CCC CAG CAG GTA CAA CG-3′ (reverse); human GAPDH 5′-ACA ACT TTG GTA TCG TGG AAG G-3′ (forward) and 5′-GCC ATC ACG CCA CAG TTT C-3′ (reverse). The qPCR parameters were set for one cycle at 95 °C for 10 min, 40 cycles at 95 °C for 15 s, and 60 to 65 °C for 1 min. GAPDH was utilized as an internal control. Following PCR, mRNA levels were analyzed using the Bio-Rad CFX Manager, version 3.1.

### Western blot analysis

For protein analysis, astrocytes (10^6^–10^7^ cells) from cultures were harvested and used for protein extraction and further analysis. Samples were homogenized in a radioimmunoprecipitation assay buffer containing protease inhibitors, followed by bicinchoninic acid assay. Thirty micrograms of protein per sample were resolved in 10% SDS-PAGE gels and transferred to a nitrocellulose membrane for Western blot analysis. The primary antibodies were used at a 1:1000 dilution, and HRP-conjugated secondary antibodies were used at a 1:5000 dilution. Protein bands were detected with SuperSignal West Pico PLUS Chemiluminescent Substrate (Thermo Scientific) and quantified using the Molecular Imager ChemiDoc XRS+ System (Bio-Rad).

### Immunocytochemistry and PLA

For immunostaining and PLA, cells were cultured on poly-l-lysine–coated cover glass slips in 6-well plates. After each experiment, cells were fixed using 4% paraformaldehyde in PBS at pH 7.4 for 10 min at room temperature; then, cells were washed three times with ice-cold PBS. Cells were permeabilized, then incubated with blocking buffer (1× PBS/10% normal serum/0.1% Tween-20) for 1 h at room temperature, washed, and incubated overnight with primary antibodies for EAAT2, REST, CBP/p300, or CREB at 1:250 dilution at 4 °C. After overnight incubation, tissue sections were incubated with fluorescent-conjugated secondary antibody Alexa Fluor 488 and 568 (1:1000 dilution) for 1 h at room temperature in the dark, then washed, and mounted on to slides with 4′,6-diamidino-2-phenylindole fluoromount solution for imaging analysis.

PLA was performed according to the manufacturer's instructions (MilliporeSigma). Briefly, after treated, cells were fixed with 4% formaldehyde in PBS, followed by incubation with primary antibodies for REST, CBP/p300, and/or CREB (1:250 dilution) at 4 °C overnight. Then, secondary antibodies conjugated with oligonucleotides were added and incubated at 37 °C for 1 h, followed by incubation with ligation solution containing two oligonucleotides and ligase at 37 °C for 30 min to hybridize two proximity oligonucleotides, which produces closed loops if the two interacting proteins are in proximity. Then, amplification solution was added to intensify the fluorescent signals of hybridized oligonucleotides to visualize red fluorescent signals. Before washing, cells were incubated with Alexa Fluor 488-conjugated secondary antibody to determine REST expression and its cellular localization in astrocytes. Cellular localization and fluorescence intensity were assessed for each sample using a Ts2R fluorescence microscope (Nikon Instruments) and a Leica SPEII confocal microscope (Leica Microsystems, Inc).

### ChIP assay

The ChIP assay was performed using the EZ-ChIP kit (MilliporeSigma) according to the manufacturer's instructions. Briefly, crosslinking was done by treating cells with formaldehyde for 10 min at room temperature. After washing with ice-cold PBS, cells were lysed in SDS lysis buffer containing protease inhibitor cocktail. The cell lysates were sonicated and centrifuged at 15,000*g* for 10 min at 4 °C. The supernatant was mixed with ChIP dilution buffer, and 60 μl of protein G agarose was added. After 1 h incubation at 4 °C, the agarose beads were pelleted by spinning at 3000*g* for 1 min. Then, 1% of the supernatants were saved as inputs, and REST or rabbit IgG (negative control) antibodies were added to the remainder and incubated overnight at 4 °C. Protein G agarose beads (60 μl) were added and incubated at 4 °C for 1 h. Finally, the agarose beads were pelleted and washed with low salt and high salt immune complex wash buffer. The free DNA obtained after reverse crosslinking of protein–DNA complex was purified. Real-time qPCR was carried out with the RE1 primer pairs for −663 site: 5′-GAG CTG AAG CGG GTG CTC-3′ (forward) and 5′-AAT TAG CCA AAT AAG AAA AGA GG-3′ (reverse); and for −131 site: 5′-TGA TGT CAG CTC TCG ACG AA-3′ (forward) and 5′-AGA GAG TGG TGG CAG AGG AC-3′ (reverse). Following qPCR, immunoprecipitated DNA was quantified using the Bio-Rad CFX Manager 3.1, and fold enrichment values were expressed as percent of input.

### DAPA

DAPA was performed according to the manufacturer's recommendation using a μMACS FactorFinder Kit (Miltenyi Biotec, Inc). Briefly, 1.5 μg of biotinylated oligonucleotides were incubated with 50 μg of nuclear extract as described previously (50) in a binding buffer for 20 min. The incubation was continued for another 10 min after the addition of 100 μl of Streptavidin microbeads. Then, the reaction mixture was applied onto the microcolumn that was already equilibrated with two 100-μl washes of binding buffer. Finally, after four washes of 100 μl each with low-salt and high-salt buffers, proteins were eluted using 30 μl of elution buffer and analyzed by Western blotting.

### EMSA

EMSA was performed using a LightShift Chemiluminescent kit (Thermo Scientific) according to the manufacturer's instructions. Briefly, 5 μg of nuclear extract from cells were incubated with biotin-labeled oligonucleotides containing REST consensus binding sites of the EAAT2 promoter for 20 min on ice. The DNA–protein complexes were resolved on 8% nondenaturing DNA polyacrylamide gels and transferred to nylon membranes. DNA–protein complexes were detected using a Chemiluminescent Nucleic Acid Detection Module (Thermo Scientific). The primers pairs used for REST (EAAT2 promoter including the RE1 consensus site) were 5′-GAG GAG GGA GCG CCA GGG GCT GCT CCA GGG A-3′ and 5′-TCC CTG GAG CAG CCC CTG GCG CTC CCT CCT C-3′ for −663 and 5′-CGC AGC AAA GCA CAG GTG GCA GCG GCT GCA G-3′ and 5′-CTG CAG CCG CTG CCA CCT GTG CTT TGC TGC G-3′ for −131 site.

### Co-IP

Co-IP was performed as described previously with slight modifications (24). Briefly, 400 μg of nuclear extracts were mixed with 2 μg of antibody, followed by incubation with 20 μl of protein A/G-agarose beads (Santa Cruz) overnight at 4 °C. Agarose beads were washed three times with radioimmunoprecipitation assay buffer, and 40 μl of 2× SDS sample buffer was added to elute the bound proteins from the beads; samples were then boiled at 95 °C for 5 min. Supernatants were collected and analyzed for Western blotting. Nuclear extracts were treated with DNase I (Sigma–Aldrich) to exclude possible DNA–protein interaction during the co-IP.

### Site-directed mutagenesis

The RE1 consensus binding sequence in the EAAT2 promoter was mutated using PfuUltra II Fusion HS DNA Polymerase Kit (Agilent Technologies) according to the manufacturer's recommendation. The EAAT2 promoter (−954 to +44 bp) subcloned into the LightSwitch promoter reporter vector was used as the original template for mutation. The primer sets used for mutation of RE1 site are at −663 (mutant 1) 5′-GGG AGC G***TT*** AGG GGC TGC TCC-3′ and 5′-GGG AGC G***T***C AGG ***T***GC TGC TCC-3′ and RE1 site at −131 (mutant 2) 5′-CAA AGC A***T***A GGT ***T***GC AGC GGC-3′ and 5′-CAA AGC A***T***A GGT GGC ***A***GC GGC-3′. The RE1 mutant clones were confirmed by Sanger sequencing.

### Assays of oxidative stress, calcium (Ca^2+^) influx, mitochondrial Δψm, apoptosis, and cell viability in astrocyte–neuron coculture model

Coculture of Mn-treated EV-/REST-transfected astrocytes and differentiated LUHMES cells were treated with toxic concentrations of glutamate (250 μM, 12 h). Endpoint product fluorescence was measured in each assay using the Spectramax i3x Multi-Mode microplate reader from Molecular Devices. To measure cell viability and apoptosis, 10 μl of resazurin (5 mg/ml in PBS) or annexin V was added, and cells were incubated for 15 min at 37 °C, followed by measurement of fluorescence intensity at excitation/emission wavelength of 530/590 nm for resazurin and 494/518 for annexin V–FITC. Ca^2+^ influx as an indicator of neuronal excitation and activation was measured using Life Technologies Fluo-4AM. Mitochondrial Δψm as an indicator of mitochondrial health and functional status was measured using the Life Technologies tetramethylrhodamine ethyl ester. Generation of ROS as an indicator of oxidative stress was measured using the Life Technologies ROS probe chloromethyl derivative of 2′,7′-dichlorodihydrofluorescein diacetate. Endpoint fluorescence was determined at an excitation/emission wavelength of 494/506 nm (for Ca^2+^ influx), 549/575 nm (for Δψm), and 485/527 nm (for ROS).

### Flow cytometry

For protein immunostaining, cells were washed twice and resuspended in cell staining buffer, and then incubated with anti-EAAT2 and anti-REST antibodies at 1:100 dilution. After incubation for 1 h, cells were washed twice, followed by staining with fluorescent-labeled secondary antibodies for 30 min. Fluorescent cells were analyzed by a BD FACSCalibur 2.0 flow cytometer using FlowJo X software (version 10).

### Statistical analysis

All data were expressed as the mean ± SD. The statistical analyses were performed using either Student's *t* test or one-way ANOVA, followed by Tukey's post hoc tests using the GraphPad Prism Software, version 6.0 (GraphPad Software, Inc). A *p* value of less than 0.05 (*p* < 0.05) was considered statistically significant.

## Data availability

All data are available within the article.

## Supporting information

This article contains supporting information.

## Conflict of interest

The authors declare no conflicts of interest with the contents of this article.
