# Supplementary material for: Astrocytic transcription factor REST upregulates glutamate transporter EAAT2, protecting dopaminergic neurons from manganese-induced excitotoxicity
Source: J Biol Chem. 2021 Oct 29;297(6):101372. doi: 10.1016/j.jbc.2021.101372 (PMC8626589; doi:10.1016/j.jbc.2021.101372)
Supplement: Supplemental Figure S1 [file mmc1.docx]

**
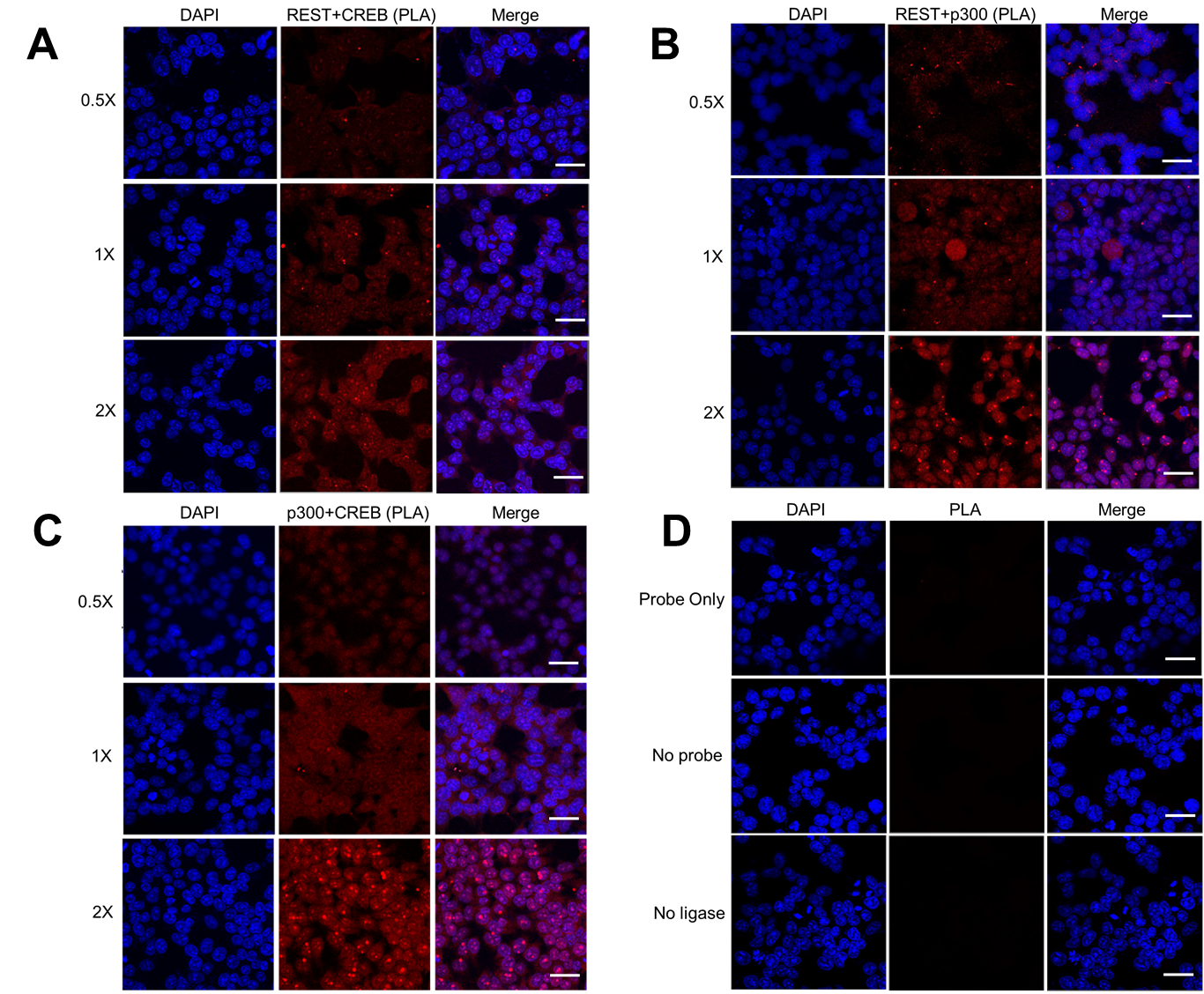
**

**Supplementary Figure 1. Validation of proximity ligation assay (PLA) in human H4 astrocytes.** Several concentrations (0.5X, 1X and 2X) of primary antibodies for REST, CREB, and CBP/p300 were tested to confirm that the PLA results are dose-dependent and validate the PLA. REST interacted with CREB and CBP/p300 in an antibody concentration-dependent manner by PLA (Suppl. Fig. 1A and B). CREB also interacted with CBP/p300 (Suppl. Fig. 1C) in H4 astrocytes as described in the Experimental Methods (×40 magnification with confocal microscope, scale: 25 µm). Negative controls such as probe only, no probe and no ligase were also tested to validate the PLA, showing no red fluorescence (Suppl. Fig. 1D).
